# Assessment of Prolonged Physiological and Behavioral Changes Associated With COVID-19 Infection

**DOI:** 10.1001/jamanetworkopen.2021.15959

**Published:** 2021-07-07

**Authors:** Jennifer M. Radin, Giorgio Quer, Edward Ramos, Katie Baca-Motes, Matteo Gadaleta, Eric J. Topol, Steven R. Steinhubl

**Affiliations:** 1 Scripps Research Translational Institute, San Diego, California; 2 Care Evolution, Ann Arbor, Michigan

## Abstract

This cohort study examines the duration and variation of recovery among COVID-19–positive verses COVID-19–negative individuals.

## Introduction

Long-term COVID symptoms marked by autonomic dysfunction^1^ and cardiac damage^2^ following COVID-19 infection have been noted for up to 6 months after symptom onset,^3^ but to date have not been quantified, to our knowledge. Previous studies have found that wearable data can improve real-time detection of viral illness^4^ or discrimination of individuals with COVID-19 vs other viral infections.^5^ Wearable devices provide a way to continuously track an individual’s physiological and behavioral metrics beginning when healthy (ie, before infection), during the course of infection, and recovery back to baseline. In this cohort study, we aimed to examine the duration and variation of recovery among COVID-19–positive vs COVID-19–negative participants.

## Methods

DETECT (Digital Engagement and Tracking for Early Control and Treatment) is a remote, app-based, longitudinal research study enrolling adult participants from all over the US and collecting their wearable data to better understand individual changes associated with viral illness, including COVID-19. All participants provided informed consent electronically. The protocol for this study was reviewed and approved by the Scripps Office for the Protection of Research Subjects. This study follows the Strengthening the Reporting of Observational Studies in Epidemiology (STROBE) reporting guideline.

From March 25, 2020, through January 24, 2021, 37 146 participants were enrolled. This analysis focuses on 875 individuals who reported symptoms of an acute respiratory illness and underwent swab testing for COVID-19 and were found to be either positive (234 individuals) or negative (641 individuals) (eFigure in the Supplement).

The following calculation was used for resting heart rate (RHR): deviation from baseline = daily RHR − baseline RHR mean. Individuals with COVID-19 were also grouped by their mean RHR deviation from baseline 28 to 56 days after symptom onset (<1, 1-5, or >5 beats per minute).

Data analysis was conducted in SAS statistical software version 9.4 (SAS Institute). Significance was set at *P* < .05. *P* values were calculated with 1-way ANOVA (for mean age) or χ^2^ tests. Additional details about our methods can be found in the eAppendix in the Supplement.

## Results

For this analysis, our study population consisted of 234 COVID-19–positive individuals (mean [range] age, 45.3 [18-76] years; 164 women [70.9%]) and 641 COVID-19–negative symptomatic individuals (mean [range] age, 44.7 [19-75] years; 455 women [71.1%]). Individuals with COVID-19 took longer to return to their RHR (Figure, A and B), sleep (Figure, C and D), and activity (Figure, E and F) baselines compared with symptomatic individuals who were COVID-19 negative. This difference was most marked for RHR, with COVID-19–positive individuals initially experiencing a transient bradycardia followed by a prolonged relative tachycardia that did not return to baseline, on average, until 79 days after symptom onset. Step count and sleep quantity returned to baseline sooner than RHR at 32 and 24 days, respectively. During recovery, individuals with COVID-19 experienced different trajectories in the return of their RHR to their normal compared with COVID-19–negative individuals (Figure, B). A small subset of COVID-19–positive participants (32 participants [13.7%]) maintained an RHR more than 5 beats per minute greater than their baseline RHR that did not return to their normal for more than 133 days. During the acute phase of COVID-19, individuals in this group reported higher frequencies of cough (27 participants [84.4%] vs 57 participants [55.3%] in the <1 beat per minute group and 57 participants [57.6%] in the 1-5 beats per minute group), body ache (20 participants [62.5%] vs 42 participants [40.8%] in the <1 beat per minute group and 35 participants [35.4%] in the 1-5 beats per minute group), and shortness of breath (9 participants [28.1%] vs 9 participants [8.7%] in the <1 beat per minute group and 6 participants [6.1%] in the 1-5 beats per minute group) compared with the other groups (Table).

**Figure.**
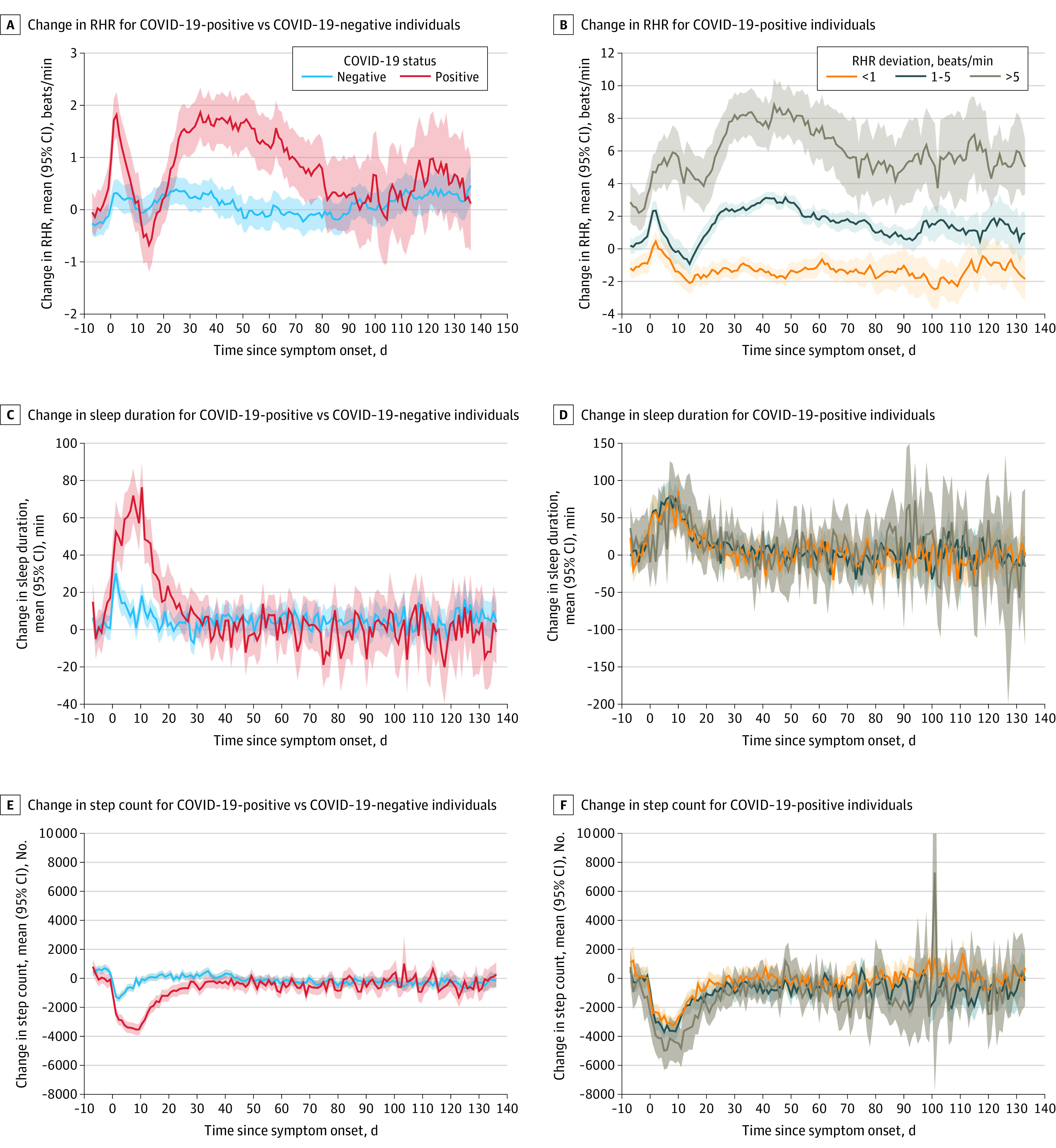
Mean Change in Wearable Data From Individual Baseline Before and After COVID-19 Symptom Onset Graphs show mean deviation from baseline (lines) with 95% CIs (shaded areas) for daily resting heart rate (RHR), sleep quantity, and step count during −7 to 133 days after symptom onset for COVID-19–positive vs COVID-19–negative participants (panels A, C, and E) and for COVID-19–positive participants grouped by mean change in RHR during days 28 to 56 after symptom onset (panels B, D, and F).

**Table.** Population Characteristics and Frequencies of Self-reported Symptoms Among COVID-19–Positive Participants Characterized by Mean RHR Change From Baseline During 28 to 56 Days After Symptom Onset

Characteristic	Patients, No. (%), mean RHR change			*P* value^a^
	<1 beat/min (n = 103)	1-5 beats/min (n = 99)	>5 beats/min (n = 32)	
Gender				
Female	75 (73.5)	71 (71.7)	18 (56.3)	.16
Male	27 (26.5)	28 (28.3)	14 (43.8)	
Age, mean (SD), y	45.3 (13.1)	45.3 (11.8)	45.4 (11.7)	>.99
Fever, chills, or sweating	39 (37.9)	32 (32.3)	14 (43.8)	.46
Temperature, °F, mean (SD)	98.5 (1.2)	98.5 (1.2)	98.7 (1.1)	.54
Cough	57 (55.3)	57 (57.6)	27 (84.4)	.01
Body ache	42 (40.8)	35 (35.4)	20 (62.5)	.03
Difficulty breathing	9 (8.7)	6 (6.1)	9 (28.1)	.001
Stomachache	9 (8.7)	2 (2.0)	0	.03
Diarrhea or vomiting	19 (9.7)	14 (14.1)	5 (15.6)	.53
Fatigue	50 (48.5)	45 (45.5)	12 (37.5)	.55
Congestion or runny nose	64 (62.1)	58 (58.6)	15 (46.9)	.31
Headache	55 (53.4)	56 (56.6)	15 (46.9)	.63
Decrease in taste or smell	26 (25.2)	27 (27.3)	9 (28.1)	.92
Sore throat	40 (38.8)	35 (35.4)	14 (43.8)	.68

Abbreviation: RHR, resting heart rate.

SI conversion factor: To convert degrees Fahrenheit to degrees Celsius, subtract 32 and multiply by 0.556.

^a^ *P* values were calculated with 1-way ANOVA (for mean age) or χ^2^ test.

## Discussion

To our knowledge, this is the first study to examine longer duration wearable sensor data. We found a prolonged physiological impact of COVID-19 infection, lasting approximately 2 to 3 months, on average, but with substantial intraindividual variability, which may reflect various levels of autonomic nervous system dysfunction or potentially ongoing inflammation. Transient bradycardia has been noted in a case study^6^ approximately 9 to 15 days after symptom onset, which was also seen in our population. Our data suggest that early symptoms and larger initial RHR response to COVID-19 infection may be associated with the physiological length of recovery from this virus.

Symptom data were collected only during the acute phase of infection, which limited our ability to compare long-term physiological and behavioral changes with long-term symptoms. In the future, with larger sample sizes and more comprehensive participant-reported outcomes, it will be possible to better understand factors associated with interindividualized variability in COVID-19 recovery.

## Notes

Supplement. **eAppendix.** Supplemental Methods **eFigure.** Flow Chart of Selecting Participants for Final Dataset eReference
