# Supplementary material for: Assessment of Prolonged Physiological and Behavioral Changes Associated With COVID-19 Infection
Source: JAMA Netw Open. 2021 Jul 7;4(7):e2115959. doi: 10.1001/jamanetworkopen.2021.15959 (PMC8264646; doi:10.1001/jamanetworkopen.2021.15959)
Supplement: Supplement. — eAppendix. Supplemental Methods eFigure. Flow Chart of Selecting Participants for Final Dataset eReference [file jamanetwopen-e2115959-s001.pdf]

Supplemental Online Content

Radin JM, Quer G, Ramos E, et al. Assessment of prolonged physiological and behavioral changes associated with COVID-19 infection. *JAMA Netw Open*. 2021;4(7):e2115959. doi:10.1001/jamanetworkopen.2021.15959

eAppendix. Supplemental Methods

eFigure. Flow Chart of Selecting Participants for Final Dataset

eReference

This supplemental material has been provided by the authors to give readers additional information about their work.

© 2021 Radin JM et al. *JAMA Network Open.*

eAppendix. Supplemental Methods

Research app

Individuals can participate in the DETECT study by downloading the research app MyDataHelps, available in Android and iOS. In addition to sharing wearable data, participants are also asked to self-report symptoms, test results, vaccines, demographic data, and share electronic health record data when possible through the app.

Sensor metrics

For this sub-study, we considered RHR, sleep, and activity data from Fitbit devices only to ensure consistency for the daily RHR calculation which is proprietary for each device. Fitbit's daily resting heart rate is closest to the measurement taken lying down before someone woke up in the morning.[1]

Statistical Analysis

Participant's "healthy" baseline was calculated by identifying their average and variation of each metric (daily resting heart rate (RHR), step count and sleep duration) >=7 days prior to symptom onset.

Wear Time

On average individuals with COVID-19 wore their device 75.4% of the time during days -7 to 133.

eFigure. Flow Chart of Selecting Participants for Final Dataset

Only participants who completed a sick survey and reported a COVID-19 PCR test result were included. If a participant had more than 1 COVID-19 test, the first reported positive COVID-19 test was selected first followed by the first negative test. Only individuals who received a COVID-19 PCR test within 0-14 days after symptom onset were included. Participants in the final dataset had to wear their device for >=7 days prior to the sick period. Individuals also had to have >= 1 RHR during -7 to 13 days post symptom onset, >=1 RHR during 15-27 days post symptom and >=1 RHR during 28-56 days post symptom onset.

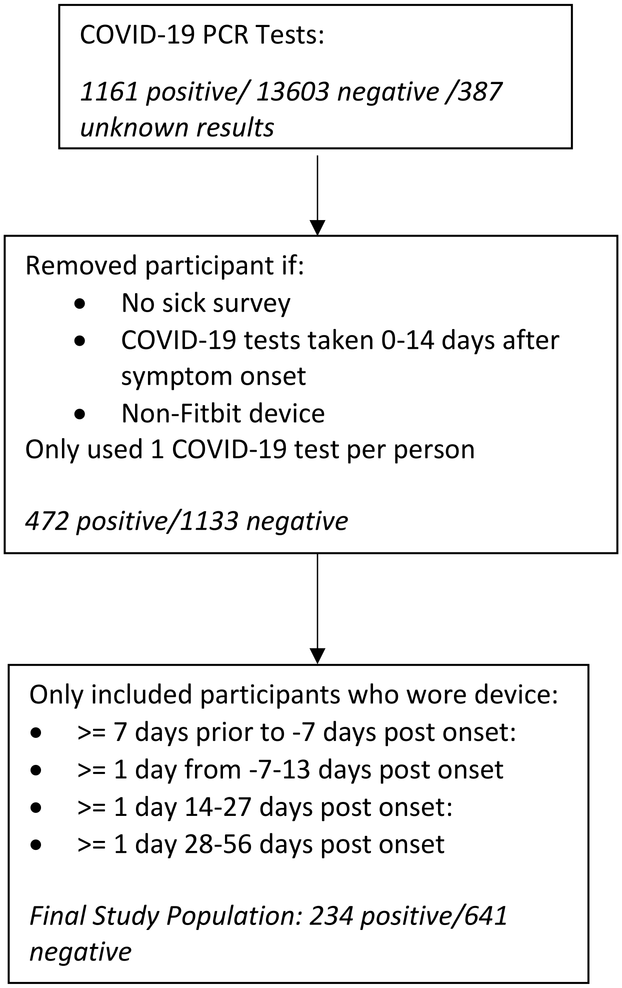

© 2021 Radin JM et al. *JAMA Network Open.*

eReference

1. Heneghan C, Venkatraman S, Russell A. Investigation of an estimate of daily resting heart rate using a consumer wearable device. *medRxiv.* 2019:19008771.

© 2021 Radin JM et al. *JAMA Network Open.*
